# Transcriptome profiles and chromatin states in mouse androgenetic haploid embryonic stem cells

**DOI:** 10.1111/cpr.13436

**Published:** 2023-03-01

**Authors:** Weisheng Zheng, Liping Wang, Wenteng He, Xinjie Hu, Qianshu Zhu, Liang Gu, Cizhong Jiang

**Affiliations:** ^1^ Key Laboratory of Spine and Spinal Cord Injury Repair and Regeneration of Ministry of Education, Orthopaedic Department of Tongji Hospital, Shanghai Key Laboratory of Signaling and Disease Research, School of Life Sciences and Technology Tongji University Shanghai China; ^2^ Institute for Regenerative Medicine, Shanghai East Hospital, School of Life Sciences and Technology Tongji University Shanghai China; ^3^ Frontier Science Center for Stem Cell Research Tongji University Shanghai China

## Abstract

Haploid embryonic stem cells (haESCs) are derived from the inner cell mass of the haploid blastocyst, containing only one set of chromosomes. Extensive and accurate chromatin remodelling occurs during haESC derivation, but the intrinsic transcriptome profiles and chromatin structure of haESCs have not been fully explored. We profiled the transcriptomes, nucleosome positioning, and key histone modifications of four mouse haESC lines, and compared these profiles with those of other closely‐related stem cell lines, MII oocytes, round spermatids, sperm, and mouse embryonic fibroblasts. haESCs had transcriptome profiles closer to those of naïve pluripotent stem cells. Consistent with the one X chromosome in haESCs, *Xist* was repressed, indicating no X chromosome inactivation. haESCs and ESCs shared a similar global chromatin structure. However, a nucleosome depletion region was identified in 2056 promoters in ESCs, which was absent in haESCs. Furthermore, three characteristic spatial relationships were formed between transcription factor motifs and nucleosomes in both haESCs and ESCs, specifically in the linker region, on the nucleosome central surface, and nucleosome borders. Furthermore, the chromatin state of 4259 enhancers was off in haESCs but active in ESCs. Functional annotation of these enhancers revealed enrichment in regulation of the cell cycle, a predominantly reported mechanism of haESC self‐diploidization. Notably, the transcriptome profiles and chromatin structure of haESCs were highly preserved during passaging but different from those of differentiated cell types.

## Graphical abstract

Schematic illustration of the extensive and accurate reprogramming making haESCs a unique cell type. Through accurate reprogramming, haESCs have a naïve pluripotency state. The characteristic spatial relationships between the motifs of transcription factors and nucleosomes are critical for acquisition and maintenance of pluripotency. The distinct nucleosome occupancy in the nucleosome depletion regions in the promoters of haESCs is associated with the role of haESCs as spermatoid stem cells. The chromatin state of the enhancers may play a role in self‐diploidization of haESCs.

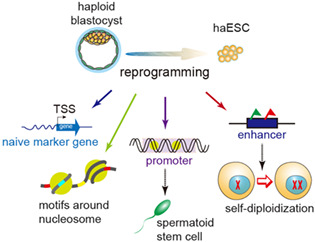

## 1 INTRODUCTION

Haploid embryonic stem cells (haESCs) comprise a unique type of ESC that contain only one set of chromosomes. Similar to ESCs, haESCs are also generated from the inner cell mass (ICM), but the ICM is from the haploid blastocyst. If one set of chromosomes is inherited from the sperm, haESCs are androgenetic (AG‐haESCs). Accordingly, parthenogenetic haESCs inherit the genome only from the oocyte.^1^ With decades of optimization of their derivation and culture, an haESC line was first successfully established from medaka fish.^2^ Then, haESC lines from mouse,^3^, ^4^ rat,^5^ monkey,^6^ and human^7^, ^8^ were established. Haploid somatic cells,^9^, ^10^ haploid epiblast stem cells,^11^, ^12^ and haploid trophoblast stem cells^13^, ^14^ were also generated. Since haESCs contain only one set of chromosomes, they have great advantages for genetic analysis. As such, they have been used as sperm to generate live semi‐cloned mice.^8^, ^15^, ^16^, ^17^, ^18^, ^19^ This suggests that haESCs are invaluable for genetic screening and mammalian‐assisted reproduction. However, these cells tend to become diploid during culture in vivo through spontaneous diploidization,^1^, ^20^ which largely restricts their applications. Interestingly, the self‐diploidization rate is lower in late‐passage haESCs than in their early‐passage counterparts. Therefore, it is important to assess the similarities and differences between early‐passage and late‐passage haESCs at the molecular level.

Both nucleosomes and histone modifications are fundamental epigenetic factors, and together, they determine chromatin structure, which plays a pivotal role in regulating many biological processes. Nucleosome positioning controls DNA accessibility, especially the conserved motifs of transcription factors (TFs), and therefore plays critical roles in transcriptional regulation.^21^ Mouse‐induced pluripotent stem cells (iPSCs) obtain pluripotency through precise nucleosome remodelling, which establishes nucleosome organization in iPSCs that is highly similar to that in ESCs.^22^ It was reported that nucleosome eviction opens promoters and facilitates the differentiation of human ESCs into neuroectodermal cells.^23^ Likewise, histone modifications regulate gene transcription and interactions by altering the chromatin structure or recruiting non‐histone proteins.^24^ A previous study showed that there is little difference in H3K4me3 and H3K27me3 modifications between human ESCs and iPSCs.^25^ Furthermore, H3K27ac and H3K9ac signals in the *cis*‐regulatory elements (promoters and enhancers) coordinate their regulatory effect on neural stem cell differentiation.^26^ Moreover, the histone modification pattern in enhancers determines lineage commitment and can reveal the differentiation potential of progeny earlier than the transcription profile.^27^, ^28^ However, the extent to which the chromatin structure of haESCs resembles that of ESCs throughout haESC derivation remains unexplored. In addition, ESCs have two pluripotent states, naïve and primed,^29^ but which pluripotent state haESCs resemble is largely unresolved. Therefore, it is important to understand the similarities and differences in gene expression and chromatin structure between haESCs and related cells, such as naïve and primed ESCs and, sperm, among others, because such differences might affect the maintenance and applications of haESCs.

It is critical to compare haESCs with oocytes, round spermatids, and sperm because haESCs are used as gametes in genetic screens and mammalian‐assisted reproduction. Therefore, in addition to different types of stem cells, oocytes, round spermatids, sperm, and mouse embryonic fibroblasts (MEFs) as controls were also included in this study. We generated the transcriptomes, genome‐wide maps of nucleosome positioning, and core histone modifications of four mouse haESC lines and round spermatids. Then, we investigated the similarities and differences in gene expression and chromatin structure between haESCs and ESCs, MEFs, round spermatids, MII oocytes, and sperm. The results revealed that the gene expression profile and the global chromatin structure of haESCs are highly similar to those of ESCs but significantly different from those of the other cell types. The transcriptome profile and the chromatin structure were found to be preserved during haESC passaging. Moreover, haESCs have a naïve pluripotency state. Specifically, the 4259 enhancers are turned off in haESCs but have an active chromatin state in ESCs and likely function in the self‐diploidization of haESCs.

## 2 MATERIALS AND METHODS

### 2.1 Cell lines and culture

Mouse androgenetic haESC lines A129‐2 and AOS‐14, derived from mouse 129Sv and C57BL/6 strains, respectively, were established previously.^17^ A129‐2 and AOS‐14 cells were collected at early passage (passages 20–30) and late passage (passages 50–70) during in vitro cell culture. We derived another two AG‐haESC lines, A129‐6 and A129‐17, from the mouse 129Sv strain as previously described.^16^, ^17^ Briefly, sperm heads were injected into enucleated oocytes via intracytoplasmic sperm injection to construct androgenetic haploid embryos. The reconstructed embryos were activated by SrCl_2_ treatment for 4–6 h and then cultured in G1 plus medium and developed to the blastocyst stage in vitro. The blastocysts were planted on feeders in 15% KSR Knockout DMEM supplemented with 2i (1 M PD0325901 and 3 M CHIR99021). AG‐haESCs were derived from the outgrowths using FACS and the haploid cells were enriched every 3–4 passages using FACS. Mouse R1 ESCs were purchased from the American Type Culture Collection and cultured in our lab. All AG‐haESCs and ESCs were cultured in an ESC medium containing 2i.

### 2.2 Collection of round spermatids from mouse testes

Bilateral testes were obtained from humanely euthanized 8–10 week old 129Sv male mice. Seminiferous tubules were collected into a 1.5 mL Eppendorf tube after release from the testicular capsule. Then, 200 μL of 0.05% trypsin–EDTA was added, and the specimen was cut with surgical scissors for 3 min into small pieces; finally, 600 μL of 10% FBS DMEM (Gibco) was added to quench the dissociation reaction. The resultant germ cell mass suspension was gently pipetted 10 times with a 1 mL pipette into single cells and filtered first with a 70 μm filter. After centrifuging at 1000 rpm for 5 min, germ cells were stained with DMEM containing 15 μg/mL Hoechest 33,342 and 2.5 μM verapamil at 37 °C for 30 min. Then the stained germ cells were resuspended in 2 mL of BD FACS buffer after centrifuging at 1000 rpm for 5 min and filtered with a 40 μm filter. Haploid round spermatids were sorted out based on cell size and DNA content using a BD FACS Aria II flow cytometer.

### 2.3 RNA‐seq and data analysis

Haploid cells at the G0/G1 phase from individual AG‐haESC lines were collected using FACS and washed with DPBS. Total RNA was extracted from each cell line using the TIANGEN RNA simple total RNA kit (DP419), and two biological replicates were performed for each sample. The RNA concentration was measured using a NanoDrop 2000 (Thermo Scientific), and the integrity of RNA was tested via AGE (agarose gel electrophoresis). cDNA was synthesized using random hexamer primers and purified using 1.0× Agencourt AMPure XP beads. Sequencing primers were added to the cDNA fragments, and libraries were generated through PCR amplification. The sequencing was performed with an Illumina HiSeq X‐Ten or NovaSeq platform using the 150 bp paired‐end protocol.

Raw reads of RNA‐seq data were first processed to trim adapter sequences and low‐quality bases using Fastp (version 0.20.1).^30^ Then, the clean reads were mapped to the mouse mm10 genome and transcriptome using hisat2 (version 2.2.1)^31^ with the parameters ‘‐‐sensitive ‐‐dta’. Only the uniquely mapped reads were retained for further analysis. Read counts of all RefSeq genes were calculated using featureCounts (version 2.0.0).^32^ The read count matrix was inputted into DESeq2 (version 1.26.0) to model the distribution of the reads and perform cross‐sample normalization. The DESeq2 normalized read counts were then calculated as reads per kilobase per million mapped reads (RPKM) for each sample. The gene expression RPKM matrix of all samples was used to perform gene expression correlation analysis, principle component analysis, and hierarchical clustering analysis. The gene expression RPKM values of biological replicates in each sample were averaged in the analysis of the expression patterns of specific gene sets.

### 2.4 MNase‐seq and data analysis

Haploid cells of mouse AG‐haESC lines A129‐2 and AOS‐14 were collected at early passage (passages 20–30) and late stage (passages 50–70) during in vitro cell culture. For each sample, ~1 × 10^7^ haploid cells were cross‐linked with 1% formaldehyde at room temperature (RT) for 8 min, and the reaction was then terminated by adding 0.125 M glycine at RT for 5 min. After cross‐linking, the nuclei of cells were isolated and suspended in 1 mL of MNase digestion buffer (10 mM Tris–HCl, pH 7.5, 15 mM NaCl, 60 mM KCl, 1 mM CaCl_2_, 0.15 mM spermine, 0.5 mM spermidine, and 1× PI (Roche, 04693132001)). The digestion reaction was performed by adding 3 μL of MNase (Micrococcal Nuclease; NEB, M0247S) at 37 °C for 25 min and then terminated by adding EDTA to a final concentration of 10 mM. Proteinase K was added at 65 °C for 2–4 h to reverse cross‐linking. Mononucleosomal DNA fragments were purified using phenol‐chloroform and examined by running AGE and then subjected to massively parallel DNA sequencing on an Illumina HiSeq X‐Ten platform using the 150 bp paired‐end protocol.

Fastp (version 0.20.1)^30^ was employed to trim adapters and low‐quality sequences in the MNase‐seq data. The clean read pairs were mapped to the mouse mm10 genome using Bowtie2 (version 2.3.5.1)^33^ with the parameters ‘‐‐no‐unal ‐‐no‐mixed ‐‐no‐discordant’. Concordantly mapped read pairs with mapq >10 were considered high‐quality reads and retained for further analysis. The midpoints of the fragments inferred by each read pair were used to calculate the global nucleosome occupancy in each 10 kb window across the genome.

The nucleosome peaks were predicted using GeneTrack (version 1.0.3).^34^ Briefly, the fragment midpoints (termed as the index) were piled up along the genome to generate the nucleosome signal; then, the signal was smoothed using a Gaussian model with sigma = 20. Lastly, the nucleosomes were detected using an excluded zone of 147 bp. To reduce false discovery rates, we filtered out predicted nucleosomes with a low read count (rc <6).

### 2.5 TF motif enrichment in nucleosome depletion regions

Based on the nucleosome occupancy around transcription start sites (TSSs), nucleosomes are absent 180 bp upstream of the TSS for many genes. If less than 30% of a nucleosome (length) overlaps with this region, such region is defined as a promoter nucleosome depletion region (NDR). The motifs enriched in a set of the promoter NDR regions were identified using the HOMER^35^ command findMotifsGenome.pl, with default parameters.

### 2.6 Analysis of spatial relationships between nucleosomes and TF binding sites

High‐confidence peaks of TF ChIP‐seq and ATAC‐seq data in mouse ESCs were retrieved from the GEO database. The accession numbers are listed in the Data Availability Statement. As the peaks were generated using mouse mm8 or mm9 genome build, we employed the UCSC tool LiftOver (https://genome.ucsc.edu/cgi-bin/hgLiftOver) to convert the coordinates of peaks to the mm10 genome build. Then, we mapped the nucleosome signals around the peak centres of each TF and open chromatin region to analyse the spatial relationships between nucleosomes and the binding sites of the TFs in mouse haESCs and ESCs.

### 2.7 Histone modification ChIP‐seq and data analysis

ULI‐NChIP‐seq technology was employed to generate histone modification profiles in AG‐haESCs, ESCs, and round spermatids as previously described.^36^ Approximately 3 × 10^4^ cells were used per reaction, and two biological replicates were performed for each sample. Raw read pairs of histone modification ChIP‐seq data were mapped to the mm10 reference genome using Bowtie2 (version 2.3.5.1)^33^ with the parameters ‘‐‐no‐unal ‐‐no‐mixed ‐‐no‐discordant’ after trimming adapters and low‐quality sequences using Fastp (version 0.20.1).^30^ PCR duplicated reads were filtered out using Picard (version 2.17.2), and only the concordantly mapped read pairs were retained for downstream analysis. The sequencing depth‐normalized signal bigwig files for all histone modifications were generated using the Deeptools (version 3.3.0) bamCoverage function with the parameters ‘‐‐normalizeUsing RPKM ‐e ‐‐centerReads ‐‐samFlagInclude 64’.

The high‐confidence peaks of each histone modification were detected using a pipeline, as previously described with some modifications.^37^ First, we detected histone modification peaks in each biological replicate and the merged sample of biological replicates using MACS2 (version 2.2.1)^38^ with respective default parameters. Second, we randomly divided the merged sample into two pseudo replicates, and detected peaks in pseudo replicates using MACS2. Finally, the high‐confidence peaks were considered the peaks found in the merged sample that also existed in the two biological replicates or the two pseudo replicates. The Pearson correlation coefficients of sequencing depth‐normalized read densities (RPKM) of biological replicates in terms of the high‐confidence peaks were calculated to show the reproducibility of biological replicates.

### 2.8 Characterization of chromatin state in promoters and enhancers

The ±1 kb regions of TSSs of all RefSeq genes were defined as promoters. The promoters overlapping with high‐confidence peaks of H3K4me3 and H3K27me3 were defined as bivalent promoters in each sample. The high‐confidence H3K4me1 peaks were defined as enhancers in each sample. To analyse the changes in the enhancer chromatin state among early‐ and late‐passage AG‐haESCs, ESCs, and round spermatids, we first merged all H3K4me1 peaks in all AG‐haESCs and ESCs, in all AG‐haESCs and round spermatids, and in early‐ and late‐passage A129‐2 and AOS‐14 cells. Then, we calculated the read densities (RPKM) of H3K27ac, H3K27me3, H3K4me1, and the input in those merged peaks. The peaks with an RPKM value of histone modification at least 1.5‐fold greater than that of the input were considered enriched, in terms of that histone modification, in each sample. The combinations of the enrichment of the three histone modifications were used to classify those peaks into five types in each sample as follows: active (H3K4me1+/H3K27ac+/H3K27me3−), poised (H3K4me1+/H3K27ac−/H3K27me3+), intermediate (H3K4me1+/H3K27ac+/H3K27me3+), primed (H3K4me1+/H3K27ac−/H3K27me3−) and off (H3K4me1−).

### 2.9 Target genes of enhancers

The target gene of a given enhancer is the gene whose TSS is closest to the enhancer within 50‐kb region.

### 2.10 Functional annotation

ClusterProfiler (version 3.14.3)^39^ was employed for gene ontology (GO) analysis of specific gene sets. The GO terms with BH‐adjusted hypergeometric test *p*‐values <0.05 were considered to be enriched.

### 2.11 Statistical analysis

Gene expression level differences between groups were analysed by Wilcoxon test. The correlation analysis was done by Pearson test. *p*‐values <0.05 were considered significant.

## 3 RESULTS

### 3.1 Mouse AG‐haESCs are transcriptionally more similar to a naïve pluripotency state

**FIGURE 1:**
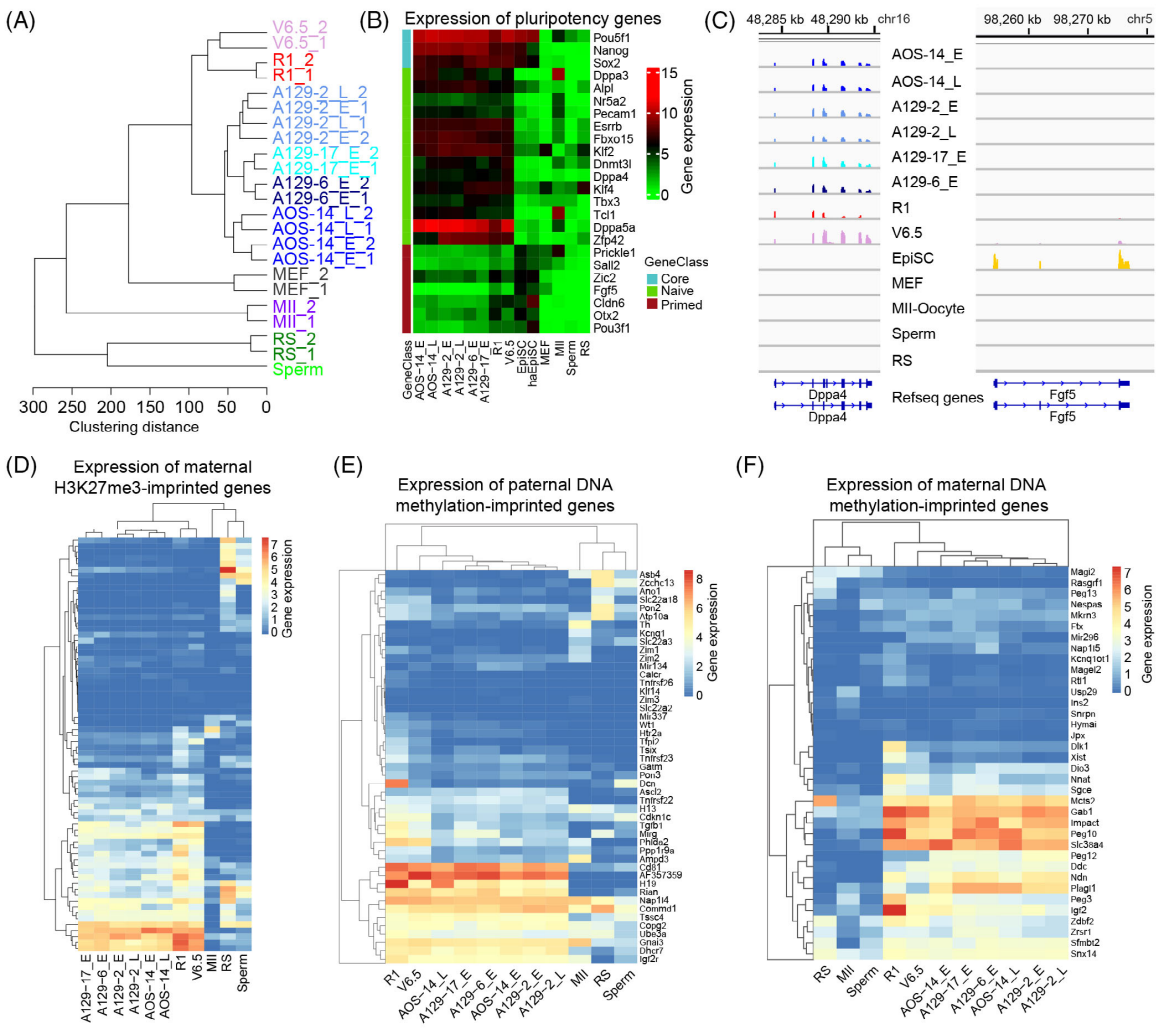
Gene expression profiling of androgenetic haploid embryonic stem cell (AG‐haESC) lines. (A) Hierarchical clustering of gene expression profiles. A129 and AOS are AG‐haESC lines. ‘E’: early‐passage (passages 20–30); ‘L’: late‐passage (passages 50–70); R1 and V6.5 are embryonic stem cells. MEFs: mouse embryonic fibroblasts; MII: MII oocytes; RS: round spermatids. All samples included two replicates, except sperm. (B) Expression levels of the core pluripotency genes, the pluripotency marker genes of naïve and primed stem cells. (C) Browser view of the expression levels of pluripotency marker genes in naïve (*Dppa4*) and primed (*Fgf5*) stem cells. (D) Expression levels of the maternal H3K27me3‐imprinted genes in the studied cell types. (E), (F) Expression levels of the paternal (E) and maternal (F) DNA methylation‐imprinted genes in the studied cell types. The gene list is given in Table S1.

We generated global gene expression profiles of four mouse AG‐haESC lines (AOS‐14, A129‐2, A129‐6, and A129‐17) and round spermatids via RNA‐seq. The transcriptome profiles between biological replicates were highly reproducible (Figure S1A). Hierarchical clustering of the gene expression profiles clustered early‐ and late‐passage AG‐haESCs and ESCs into two groups consisting of a clade that deviated from MEFs, MII oocytes, and sperm (Figure 1A). The marker genes of the three germ layers were silent or had a negligible expression level in AG‐haESCs and ESCs (Figure S1B–D). Furthermore, the marker genes for oocytes and sperm were also repressed in AG‐haESCs (Figure S1E). This indicates that the cell identity of AG‐haESCs is pluripotent stem cells rather than haploid gametes from the perspective of the transcriptome profile.

There are two distinct pluripotency states in pluripotent cells, the naïve pluripotency and primed pluripotency state. Mouse ESCs are thought to be in a naïve pluripotency state, whereas mouse epiblast stem cells (EpiSCs) are in a primed pluripotency state.^40^ To resolve the pluripotency state of AG‐haESCs, we examined expression levels of the core pluripotency marker genes and the pluripotency marker genes for naïve and primed stem cells determined in a previous study.^41^ The core pluripotency marker genes (*Pou5f1*, *Nanog*, and *Sox2*) were highly expressed in all pluripotent stem cells. Intriguingly, the pluripotency marker genes for naïve stem cells were expressed in AG‐haESCs and naïve ESCs (R1 and V6.5). In contrast, the pluripotency marker genes for primed stem cells are only expressed in the primed stem cells (EpiSCs) and haploid epiblast stem cells (haEpiSCs) (Figure 1B, C). This suggests that AG‐haESCs are transcriptionally more similar to the naïve pluripotency state.

Maternal H3K27me3 imprinting is a newly discovered type of imprinting independent of DNA methylation. The chromosomes of AG‐haESCs originate from sperm, which lack canonical histones. We then tested the expression levels of the maternal H3K27me3‐imprinted genes in AG‐haESCs. We found that approximately 40% of the 76 maternal H3K27me3‐imprinted genes^42^ were expressed in mouse AG‐haESCs and ESCs but were silenced or weakly expressed in MII oocytes and sperm (Figure 1D). Similarly, we examined the expression patterns of DNA methylation‐imprinted genes. The results showed that approximately half of maternal and paternal imprinted genes were expressed in AG‐haESCs and ESCs (Figure 1E, F). Together, these findings imply that much of the parental imprinting was lost in AG‐haESCs during in vitro culture. This is consistent with a previous report showing that parental DNA methylation imprinting is rapidly lost during in vitro cell culture.^17^, ^43^ However, increasing the cell passage number did not result in the additional loss in parental imprinting (Figure 1D–F).

One of the two X chromosomes is inactivated in female mammalian genomes.^44^ We then wondered about the scenario in haESCs with only one X chromosome. Intriguingly, the long noncoding gene *Xist*, critical for X chromosome inactivation, was repressed in haESCs (Figure S1F). This is consistent with the results of a previous study showing that *Xist* is not expressed in haploid ESCs. However, silencing *Xist* was not found to improve the maintenance of a haploid genome.^45^ Taken together, there is no need for X chromosome inactivation in haESCs, likely because there is only one X chromosome in these cells. Furthermore, X chromosome activation supplies an epigenetic signature of naïve pluripotency.^29^, ^46^ This further suggests that haESCs have a transcription profile more similar to that of naïve pluripotent stem cells.

### 3.2 Nucleosome organization of AG‐haESCs is conserved during passaging and highly similar to that of ESCs

**FIGURE 2:**
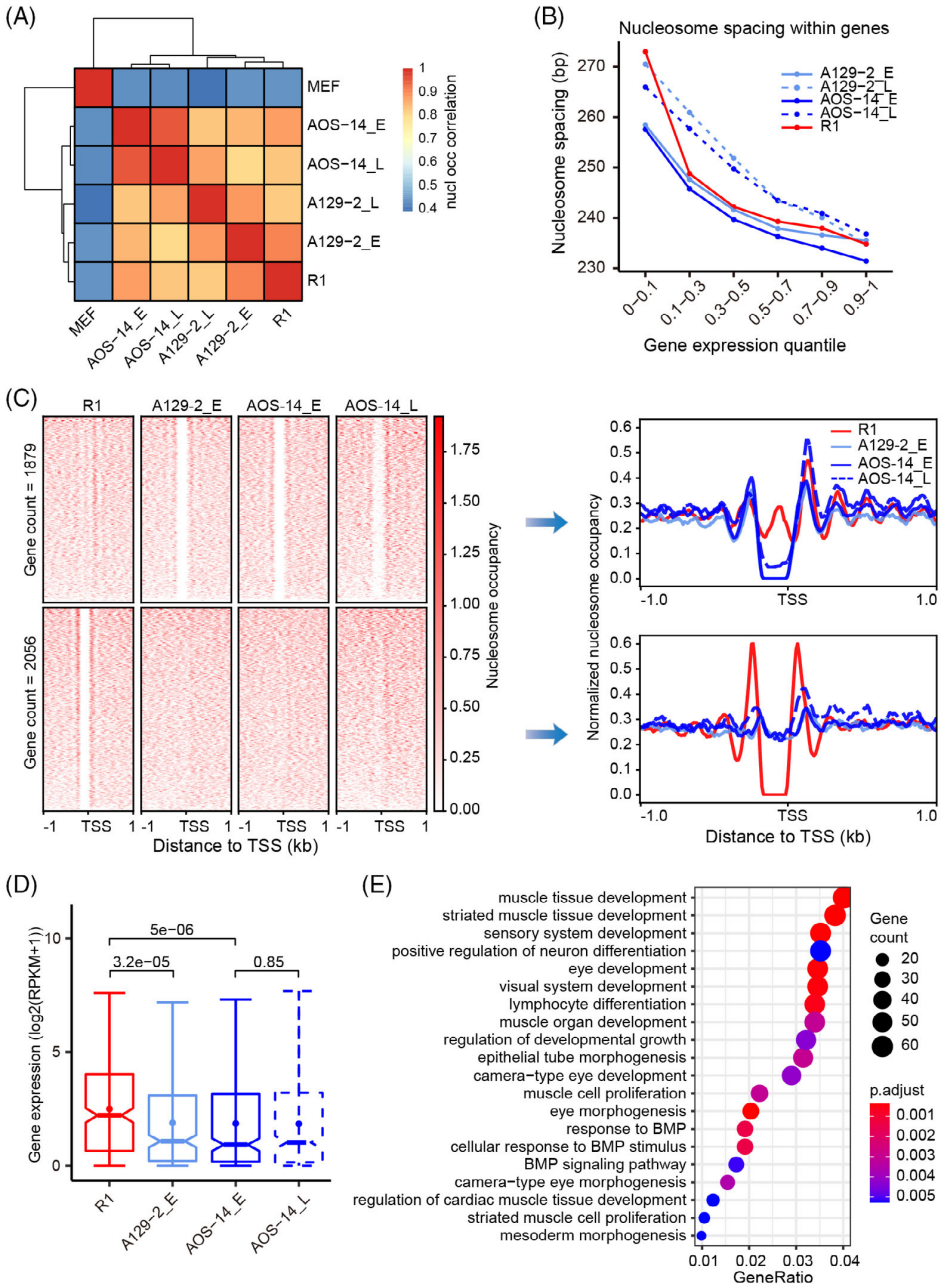
Similar global nucleosome occupancy with different local nucleosome depletion levels between androgenetic haploid embryonic stem cells (AG‐haESCs) and ESCs. (A) Pairwise correlation of global nucleosome occupancy between different cell types. (B) Nucleosome spacing in the gene body was negatively correlated with the gene expression level. (C) Heatmaps showing the nucleosome occupancy around NDRs in promoter regions that were absent in ESCs (R1) but present in AG‐haESCs (top), and vice versa (bottom). Curve plots showing the composite distribution of nucleosome occupancy around NDRs in the left heatmaps. NDR: nucleosome depletion region. (D) Expression levels of the 2056 genes in (C) (Wilcoxon test). (E) Functional annotation of the 2056 genes in (C).

We previously demonstrated that iPSCs obtained a nucleosome organization indistinguishable from that of ESCs through accurate remodelling.^22^ We then questioned the scenario in AG‐haESCs that are derived from a haploid genome. To address this, we generated genome‐wide nucleosome positioning maps of AG‐haESCs at an of single‐nucleosome resolution using MNase‐seq. Nucleosomes are prevalent throughout the entire genome. Therefore, we scanned the genome with a 10 kb window and calculated the nucleosome occupancy in each window. This nucleosome occupancy comparison showed a high correlation between AG‐haESCs and ESCs but a low correlation between this pluripotency stem cells and MEFs. Of note, the correlation between early‐ and late‐passage AG‐haESCs, in terms of nucleosome occupancy, was highest (Figure 2A). These results suggest that although they are induced from a haploid genome, AG‐haESCs obtain global nucleosome occupancy highly similar to that of ESCs, which is preserved during passaging.

Nucleosome spacing is linked to cell states and gene expression levels.^47^, ^48^ To investigate the nucleosome spacing in AG‐haESCs, we predicted the nucleosome peaks in each sample using GeneTrack^34^ and then defined the nucleosome spacing as the number of base pairs between two adjacent nucleosome midpoints. The linker DNA is the DNA between two adjacent nucleosome cores. We classified the linker DNA length into five groups based on the number of nucleosomes that the linker DNA length can hold. The distribution of the linker DNA length was similar between AG‐haESCs and ESCs. As expected, more than 75% of the linker DNA was shorter than nucleosomal DNA (~ 147 bp), and ~ 20% can hold a nucleosome. Interestingly, the number of linker DNA sites with the length required to hold a nucleosome was increased in late‐passage AG‐haESCs compared to that in early‐passage AG‐haESCs (Figure S2A). Further analysis showed that nucleosome spacing within the gene body was negatively correlated with gene expression levels. Consistent with the increase in the linker DNA length in late‐passage AG‐haESCs, nucleosome spacing in the gene body with the same expression level was larger in late‐passage AG‐haESCs than in early‐passage AG‐haESCs (Figure 2B). This suggests that nucleosome positioning overall becomes loose in the gene body during AG‐haESC passaging.

The nucleosome positioning pattern around TSSs is critical for gene expression.^49^, ^50^ We then examined the nucleosome arrangement around TSSs and observed the canonical nucleosome organization of −1, NDR, +1, +2, +3, and so forth nucleosomes around TSSs in AG‐haESCs, which is conserved during passaging and similar to that in ESCs (Figure S2B). NDRs control DNA accessibility and play an important role in cell identity.^23^, ^26^ Although global nucleosome occupancy is similar in AG‐haESCs and ESCs, they belong to different cell types. Thus, we attempted to compare NDR differences in promoter regions between AG‐haESCs and ESCs. Indeed, we identified 1879 genes for which NDRs were absent in ESCs (R1), as compared to those in AG‐haESCs, and 2056 genes that showed the opposite trend (Figure 2C). The expression of the genes with nucleosome occupancy in the NDRs in promoter regions was significantly lower than that in genes without nucleosome occupancy in the promoter NDRs (Figures 2D and S2C). Promoter NDR differences from ESCs and their effect on gene expression were found to be preserved during passaging (Figures 2C, D, and S2C). GO analysis of the 1879 genes revealed the enrichment for RNA transaction‐related functions, such as RNA metabolic process, RNA processing, and RNA modification, among others (Figure S2D). In contrast, the 2056 genes were found to be enriched for development‐ and cell proliferation‐related GO terms (Figure 2E).

We further investigated the TF motif enrichment in these two groups of NDRs. The results showed that the two sets of NDRs shared most motif enrichment. However, there were some TF motifs specifically enriched in the NDRs present in haESCs but absent in R1, such as STAT, LHX, NPAS, and bHLHE protein families (Figure S2E). STAT protein family act as transcription activators and plays a key role in cell growth and apoptosis.^51^, ^52^ It has been reported that cell death triggered by apoptosis‐related genes played a critical role in maintaining haESC haploidy.^53^, ^54^ Thus, STAT protein family may contribute to haESC haploidy. Surprisingly, LIM homeobox (LHX) proteins and neuronal PAS domain (NPAS) proteins play a regulatory role in nerve development and memory. The possible functions of these TFs in maintaining the molecular features of haESCs require more study.

### 3.3 Characteristic spatial relationship between distinct TF target sites and nucleosomes in AG‐haESCs

**FIGURE 3:**
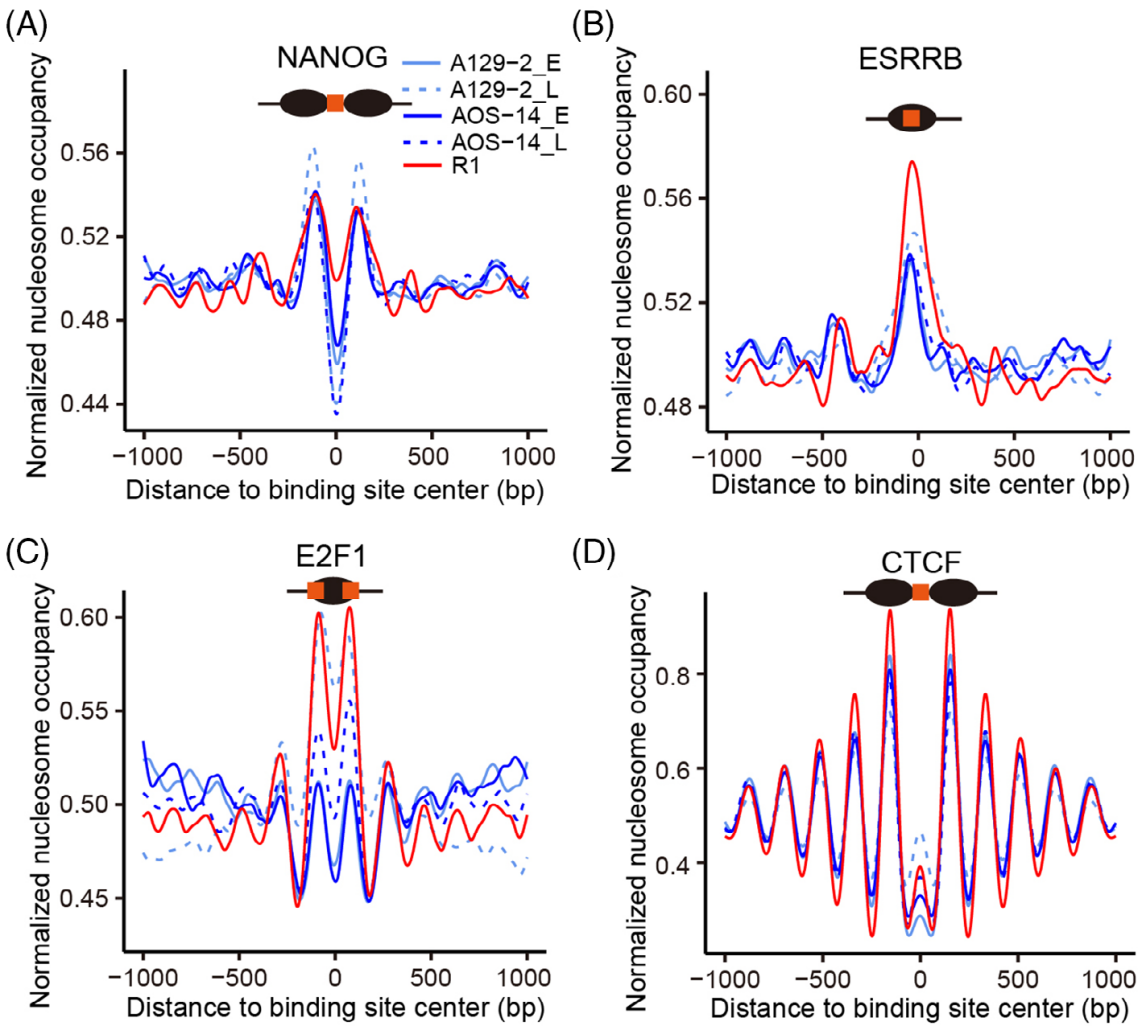
Nucleosome distribution around the binding sites of representative factors. (A) The binding sites of the core pluripotency factor NANOG were found to preferentially reside in the linker region. (B) The binding sites of the self‐renewal regulator ESRRB were predominantly present on nucleosomes. (C) The binding sites of the cell‐cycle regulator E2F1 were enriched at nucleosome borders. (D) The binding sites of the insulator CTCF were located in the linker region with a regular periodicity.

The pluripotency networks consisting of different types of TFs are important to the establishment and maintenance of the pluripotency of stem cells. Nucleosome positioning regulates the accessibility of TF motifs through translational and rotational settings.^21^ As expected, nucleosomes appeared to be distributed around open chromatin regions with a regular periodicity based on the nucleosome size (Figure S3A). That is, open chromatin regions were found in the linker regions. This sets up a translational setting for motifs. Both the core pluripotency TFs OCT4, NANOG, and SOX2 and the auxiliary TFs KLF4, E2F1, and ESRRB are critical for the maintenance of pluripotency. The epigenetic landscape often controls the binding of TFs to their target sites and therefore determines cell identity. We then explored the spatial relationship between nucleosomes and the motifs of these TFs and chromatin regulators. The binding sites for the component TFs of the core pluripotency network, for example, NANOG, OCT4, and SOX2, preferentially resided in the linker region (Figures 3A and S3B). This makes it easy for these core pluripotency TFs to bind to their target sites and establish and maintain pluripotency. Interestingly, the motifs of the core pluripotency TF KLF4 were significantly present on nucleosomes instead (Figure S3C). The binding sites of the self‐renewal regulator (ESRRB) were predominantly located on nucleosomes (Figure 3B). Interestingly, the binding sites of chromatin remodelling factors (BRG1 and HDAC1) were also enriched on nucleosomes (Figure S3D). These factors can access the motifs on nucleosomes through nucleosome rotation. Surprisingly, the binding sites of the cell‐cycle regulator E2F1 were predominantly present on the borders of nucleosomes (Figure 3C). In contrast, nucleosomes were found to be distributed around CTCF‐binding sites with a regular periodicity based on the nucleosome size (Figure 3D). It has been reported that the same nucleosome positioning occurs around CTCF‐binding sites in mouse iPSCs and human CD4+ T cells.^22^, ^55^ This indicates that CTCF mainly binds to the linker region in the immediate proximity of a nucleosome to prevent repressive chromatin regions from extending into active regions as an insulator.^55^ Collectively, nucleosome organization was found to be accurately remodelled during AG‐haESC induction such that the target sites of different TFs and chromatin remodelling factors, among others, have a characteristic spatial relationship with the nucleosome, which could contribute to the establishment and maintenance of pluripotency in AG‐haESCs. These topological relationships between motifs and nucleosomes were determined to be similar in AG‐haESCs and ESCs and preserved during passaging.

### 3.4 The H3K4me3 and H3K27me3 landscape in promoters is similar in AG‐haESCs and ESCs

**FIGURE 4:**
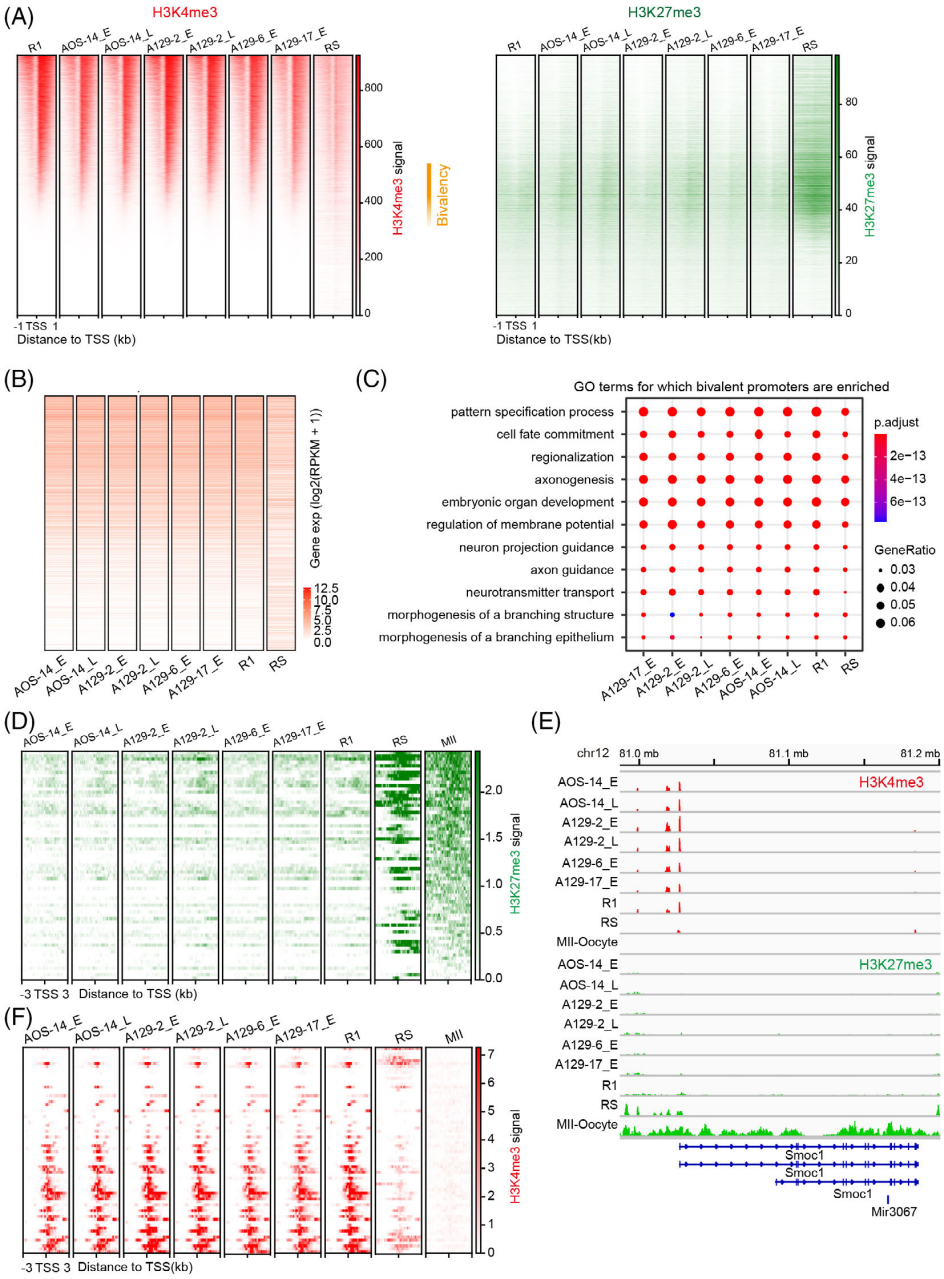
The H3K4me3 and H3K27me3 landscape in promoters is similar between androgenetic haploid embryonic stem cells (AG‐haESCs) and ESCs. (A) Heatmaps showing H3K4me3 (left) and H3K27me3 (right) signals around the 1 kb regions flanking the transcriptional start site (TSS). The regions are ordered by H3K4me3 signal levels in ESC R1. The regions in other cell types are in the same order as that for R1. H3K27me3 signals are plotted in the regions in the same order as that for H3K4me3. Bivalency indicates the regions containing both H3K4me3 and H3K27me3 in ESC R1 and AG‐haESCs. (B) Expression levels of the genes in the same order as that in (A). (C) Functional annotation of the bivalent genes in AG‐haESCs in (A). (D) H3K27me3 signals around 3 kb regions of the maternal H3K27me3‐imprinted genes in the same order as that in Figure 1D.(E) Browser view of H3K27me3 signals in the representative maternal H3K27me3‐imprinted gene *Smoc1*. (F) H3K4me3 signals around 3‐kb regions of the maternal H3K27me3‐imprinted genes in the same order as that in Figure 1D.

Histone modifications determine the chromatin state and recruit non‐histone factors to regulate gene expression. H3K4me3 and H3K27me3 are two key histone modifications, which are markers of an active and repressive state, respectively, in promoter regions. We generated ChIP‐seq data with high reproducibility to profile the histone modification landscape of AG‐haESCs, ESCs, and round spermatids (Figure S4A). The distribution of both H3K4me3 and H3K27me3 signals was highly similar between AG‐haESCs and ESCs (Figures 4A and S4B). In contrast, the H3K4me3 signal was markedly diminished, whereas the H3K27me3 signal was much higher, in round spermatids (Figure 4A). The correlations of both H3K4me3 and H3K27me3 signals were low between round spermatids and stem cells (AG‐haESCs and ESCs) (Figure S4C, D). A group of promoters in a bivalency state was also identified, that is, containing both H3K4me3 and H3K27me3 signals. There were more bivalent promoters in round spermatids. Furthermore, H3K4me3 and H3K27me3 signals appeared to be mutually exclusive in the rest of promoters (Figure 4A). Of note, both the H3K4me3 and H3K27me3 landscapes in promoter regions of AG‐haESCs were preserved during passaging (Figure S4E). As expected, gene expression levels were positively correlated with H3K4me3 signals in promoters (Figure 4B). Functional annotation of the bivalent genes in AG‐haESCs identified the enrichment for development or differential potential related GO terms, such as pattern specification process, cell fate commitment, and embryonic organ development, among others (Figure 4C). This indicates that bivalent genes often play a role in development or cell lineage commitment.

We observed that a fraction of maternal H3K27me3‐imprinted genes were activated in pluripotent stem cells (Figure 1D). To resolve the possible effect of histone modifications on the gene expression, we examined H3K27me3 and H3K4me3 signals in the promoters of these genes. Surprisingly, H3K27me3 signal levels are lower in this group of maternal H3K27me3‐imprinted genes than in the rest of the genes (Figure 4D, E). In contrast, H3K4me3 signals were also higher in this group of maternal H3K27me3‐imprinted genes (Figure 4F). This suggests that H3K27me3 loss and H3K4me3 gain likely to result in the expression of this group of maternal H3K27me3‐imprinted genes in AG‐haESCs and ESCs.

### 3.5 Distinct enhancer states in AG‐haESCs might be associated with self‐diploidization

Enhancers are important *cis*‐regulatory elements involved in cell identity determination through long‐range interactions with promoters. Histone modifications control the chromatin state of enhancers in a tissue‐specific manner. We predicted enhancers with highly reproducible histone modification ChIP‐seq data (Figure S5A). Histone modification signals in the enhancers in AG‐haESCs were highly similar to those in ESCs but different from those in round spermatids (Figure S5B, C). Histone modification landscapes in the enhancers were also found to be preserved during AG‐haESC passaging (Figure S5D, E).

**FIGURE 5:**
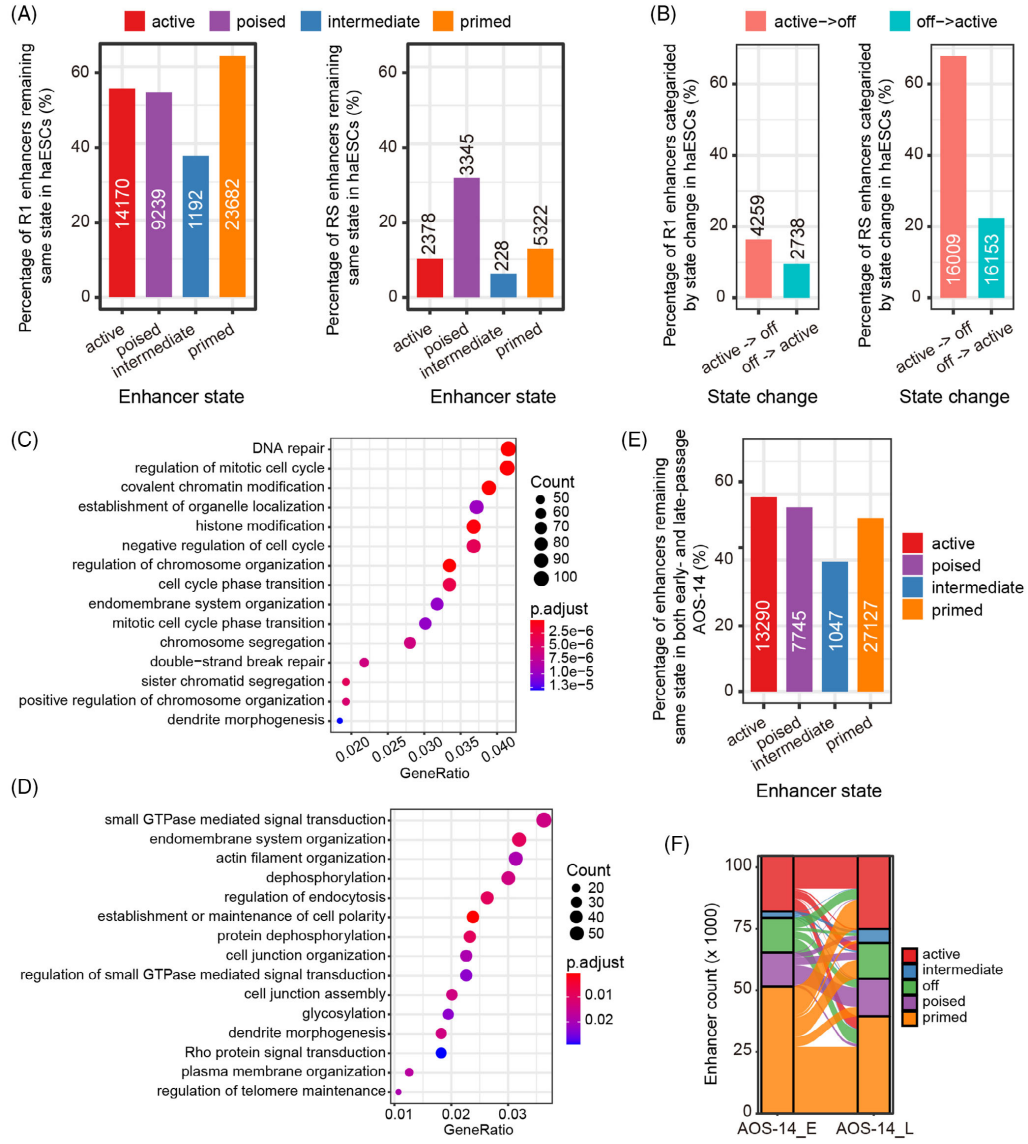
Chromatin states of enhancers. (A) Statistics of each category of enhancers in ESC R1 (left) and round spermatids (RS) (right) retaining the same chromatin state as that in androgenetic haploid embryonic stem cells (AG‐haESCs). The number of enhancers is indicated. (B) Statistics of active (red)/off (blue) enhancers in ESC R1 (left) and RS (right) changing to off/active enhancers in AG‐haESCs. The number of enhancers is indicated. (C) Gene ontology (GO) terms for which the enhancers were enriched, whose chromatin state changed from active in ESC R1 to off in AG‐haESCs. (D) GO terms for which the enhancers are enriched, whose chromatin state changed from off in ESC R1 to active in AG‐haESCs. (E) Statistics of each category of enhancers remaining in the same chromatin state during AG‐haESC passaging. The number of enhancers is indicated. (F) Alluvial plot showing the detailed change in the chromatin state of each category of enhancers during AG‐haESC passaging.

We further categorized the enhancers into different chromatin states based on histone modifications as follows: active (H3K4me1+/H3K27ac+/H3K27me3−), poised (H3K4me1+/H3K27ac−/H3K27me3+), intermediate (H3K4me1+/H3K27ac+/ H3K27me3+), primed (H3K4me1+/H3K27ac−/H3K27me3−) and off (H3K4me1−). Intriguingly, approximately 60% of the enhancers in ESC R1 had the same chromatin states as those in AG‐haESCs. In contrast, only 5%–30% of the enhancers in round spermatids had the same chromatin states as those in AG‐haESCs (Figure 5A). There were 4259 enhancers active in ESC R1 but off in AG‐haESCs, as well as 2738 enhancers showing the opposite pattern. However, 16,009 enhancers were active in round spermatids but off in AG‐haESCs, with 16,153 enhancers exhibiting the opposite trend (Figure 5B). Further functional annotation of the 4259 enhancers revealed the enrichment for GO terms related to essential cell biological processes, such as DNA repair, regulation of cell cycle, regulation of chromosome organization, chromosome segregation, and sister chromatid segregation, among others (Figure 5C). Coincidently, it has been reported that chromosome segregation and sister chromatid segregation biological processes are associated with the self‐diploidization of haESCs in cell culture.^17^, ^53^, ^56^ These abnormal enhancer states might partially explain the self‐diploidization of haESCs during passaging. GO analysis of the 2738 enhancers further identified the enrichment for a variety of functions, such as small GTPase‐mediated signal transduction, and endomembrane system organization, among others (Figure 5D).

Finally, we compared the chromatin state of enhancers between early‐ and late‐passage AG‐haESCs. The results showed that 40%–60% of each category of enhancers remained in the same state during passaging (Figure 5E). A closer examination revealed that primed and active enhancers were the top two most frequently identified enhancers (~ 70% together) in both early‐ and late‐passage AG‐haESCs. Moreover, the off enhancers in early‐passage AG‐haESCs became active, poised, and primed relatively consistently in late‐passage AG‐haESCs. In contrast, more than half of off enhancers in late‐passage AG‐haESCs originated from the primed enhancers in early‐passage AG‐haESCs (Figure 5F).

## 4 DISCUSSION

haESCs have been shown to have pluripotency with broad developmental potential and have been used as sperm replacements to ‘fertilize' oocytes and generate animal models.^8^, ^9^, ^15^, ^16^ Therefore, haESCs offer great promise for genetic analysis and assisted reproduction. However, they contain only one set of chromosomes. The extent to which haESCs resemble ESCs and differ from sperm and other cell types remained undetermined from the perspective of gene expression and the chromatin structure. We investigated the similarities and differences in gene expression and chromatin structure between mouse haESCs and other cell types. The results showed that haESCs and ESCs share key features of naïve pluripotency, which significantly differ from those of MEFs, round spermatids, sperm, and MII oocytes. Furthermore, the small variations in the chromatin structure might confer a spermatid role to haESCs and could also be involved in self‐diploidization during in vitro culture. Collectively, haESCs are a unique subtype of ESCs with naïve pluripotency.

Nucleosome positioning and histone modifications are two important factors that determine the chromatin structure, which distinguishes pluripotent ESCs from MEFs.^25^ As in iPSC generation, haESCs re‐establish the characteristic chromatin structure shared by pluripotent ESCs via accurate chromatin remodelling during their derivation. However, unlike iPSCs, AG‐haESCs inherit one set of chromosomes from sperm. It is well known that sperm lack canonical histones and concomitant histone modifications. Thus, how chromatin remodelling occurs after a sperm head is injected into an enucleated oocyte followed by haESC derivation, including protamine‐histone replacement, nucleosome assembly, and arrangement, and histone modification deposition, among other processes, remains enigmatic. Further, H3K27me3‐dependent maternal imprinting is also successfully established in haESCs. The maternal transcripts and proteins stored in oocytes are likely indispensable for chromatin remodelling. Further study is essential to identify the key factors and reveal the mechanisms associated with this.

Spontaneous diploidization during passaging in vitro results in the reversion of haESCs to diploidy, which makes it difficult to maintain haESCs over time and greatly restricts their applications. Previous studies found that cell death triggered by apoptosis‐related genes plays a critical role in maintaining haESC haploidy. The knockout of *P53* or *P73* could sustain the haploid state of haESCs.^14^, ^53^, ^54^ It was also reported that autophagy is also involved in the self‐diploidization of haESCs.^57^ Our previous finding indicated that the overexpression of *Dnmt3b* can reduce the self‐diploidization of AG‐haESCs.^17^ Interestingly, *Etl4* deficiency facilitates the stability of haESC haploidy by regulating energy metabolism.^58^ Collectively, sophisticated mechanisms underlie the self‐diploidization of haESCs. Of note, P53 also plays an essential role in regulating the cell cycle.^59^, ^60^ Therefore, an abnormal cell cycle is a predominant mechanism of diploid conversion in haESCs, including failed cytokinesis, endomitosis,^56^, ^61^ and mitotic slippage.^62^ Consistently, our results reveal that 4259 enhancers are in an off state in haESCs but active in ESCs. The functional annotation of these enhancers identified enrichment for regulation of mitotic cell cycle, chromosome segregation, and sister chromatic segregation, among others. This indicates that these enhancers likely function in the self‐diploidization of haESCs. This finding provides a new potential epigenetic means to reduce the self‐diploidization of haESCs and facilitate their applications. Nevertheless, more studies are needed to achieve this goal.

## 5 CONCLUSIONS

We unveiled the intrinsic transcriptome profile and chromatin structure of haESCs as a unique type of stem cell. haESCs have a gene expression profile more similar to that of naïve pluripotent stem cells. Because they have one X chromosome, no X chromosome inactivation occurs in haESCs. Although the chromatin structure is globally similar between haESCs and ESCs, the distinct chromatin state in thousands of *cis‐*regulatory elements (promoters and enhancers) might confer the role of spermatoid stem cells to haESCs and make them more prone to self‐diploidization during culture in vitro.

## AUTHOR CONTRIBUTIONS

Cizhong Jiang conceived the study. Weisheng Zheng and Liping Wang did most data analyses with the help from Qianshu Zhu. Wenteng He and Xinjie Hu cultured the cells and prepared the samples. Cizhong Jiang and Liang Gu supervised the study. Weisheng Zheng and Cizhong Jiang wrote the manuscript. All authors read and approved the final manuscript.

## FUNDING INFORMATION

This work was supported by the National Key R&D Program of China (2019YFA0110000 and 2021YFA1100300), the National Natural Science Foundation of China (31972882, 31721003, 31771419, and 31801206), the major project in the basic research field of Shanghai Science and Technology Innovation Action Plan (22JC1402300) and China Postdoctoral Science Foundation (2018 M640420).

## CONFLICT OF INTEREST STATEMENT

The authors have declared no conflict of interest.

## Supporting information


**Data S1:** Supporting Information

Table S1:

## Data Availability

Raw data of RNA‐seq, MNase‐seq and ChIP‐seq for haESCs and round spermatids generated in this study have been deposited in the Genome Sequence Archive of the Beijing Institute of Genomics (BIG) Data Centre with accession numbers CRA007456 (RNA‐seq), CRA007454 (MNase‐seq) and CRA007402 (ChIP‐seq). The public sequencing data are from Gene Expression Omnibus data base with accession numbers: RNA‐seq for ESC lines R1 (GSM1136063, GSM1136064) and V6.5 (GSM2417205, GSM2417217), EpiSC (GSM1382098, GSM1382099), haEpiSC (GSM5005248, GSM5005248), MEF (GSM2417196, GSM2417197), MII (GSM2588668, GSM2588669), sperm (GSM1202737); MNase‐seq for MII (GSE40896); and H3K4me3 (GSM2082662, GSM2082663) and H3K27me3 (GSM2082666, GSM2082667) ChIP‐seq for MII; ATAC‐seq for R1 (GSM2417076); protein factor ChIP‐seq for R1 NANOG (GSM2417187), OCT4 (GSM2417142), SOX2 (GSM2417143), KLF4 (GSM2417144), ESRRB (GSM2417188), E2F1 (GSM288349), CTCF (GSM288351), BRG1 (GSM2417177), HDAC1 (GSM2417173). MNase‐seq for R1 (SRA075331) is from DNA Data Bank of Japan.
