# Supplementary material for: Transcriptome profiles and chromatin states in mouse androgenetic haploid embryonic stem cells
Source: Cell Prolif. 2023 Mar 1;56(9):e13436. doi: 10.1111/cpr.13436 (PMC10472531; doi:10.1111/cpr.13436)
Supplement: Supplementary file 1 — Data S1: Supporting Information [file CPR-56-e13436-s001.docx]

Supplementary Figures and Legends:


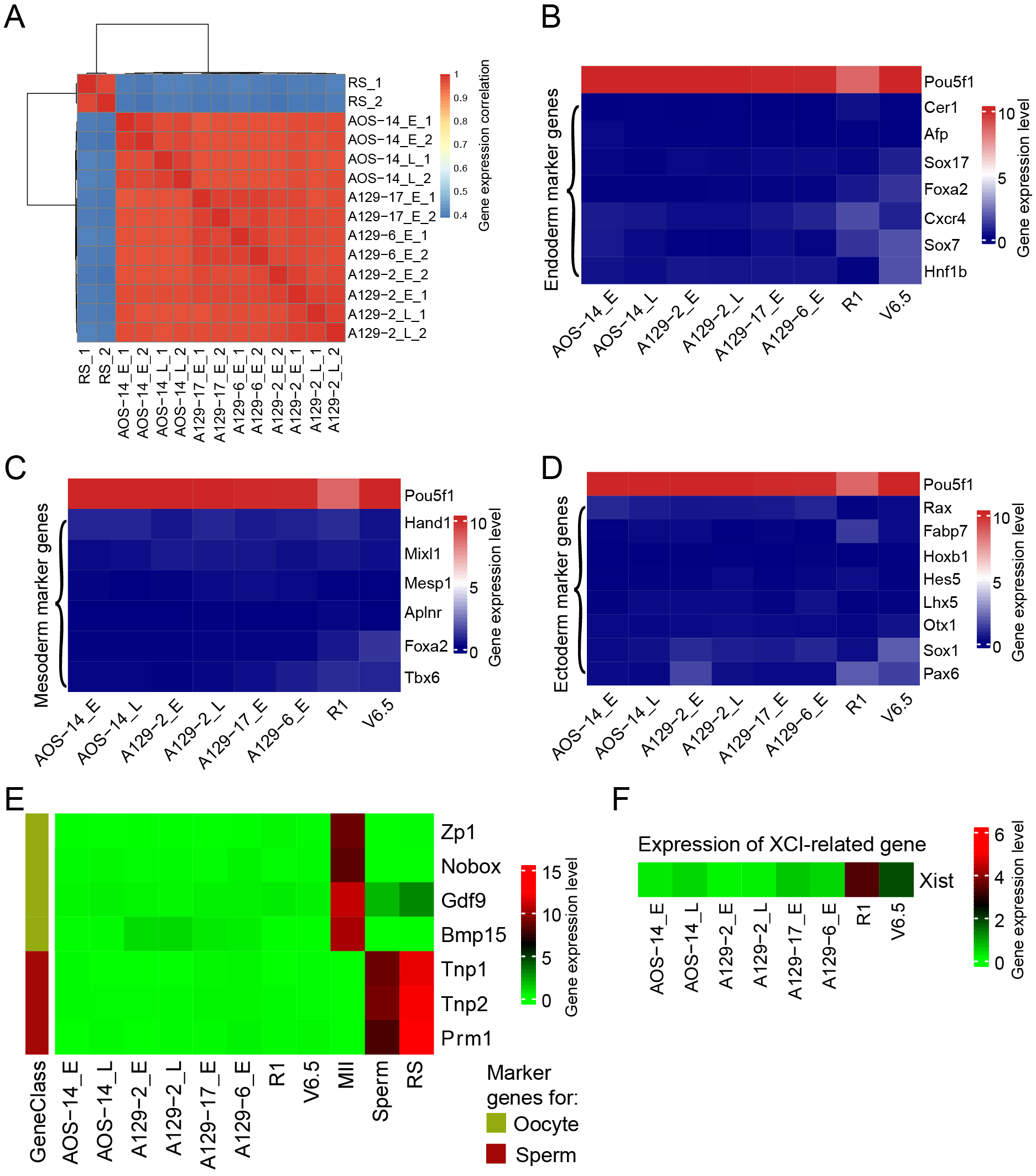


**Figure S1** Correlation of RNA-seq data and expression levels of representative marker genes.

A) The transcriptome data is highly reproducible. Correlations are also high between different AG-haESC lines, but low between AG-haESC lines and round spermatids (RS). (Pearson test)

B-D) Expression levels of the marker genes of the three germ layers in AG- haESC and ESC lines. *Pou5f1* is a highly expressed gene for comparison.

E) Expression levels of marker genes for oocyte [1] and sperm (from database CellMarker).

F) Expression levels of XCI-related gene *Xist*.


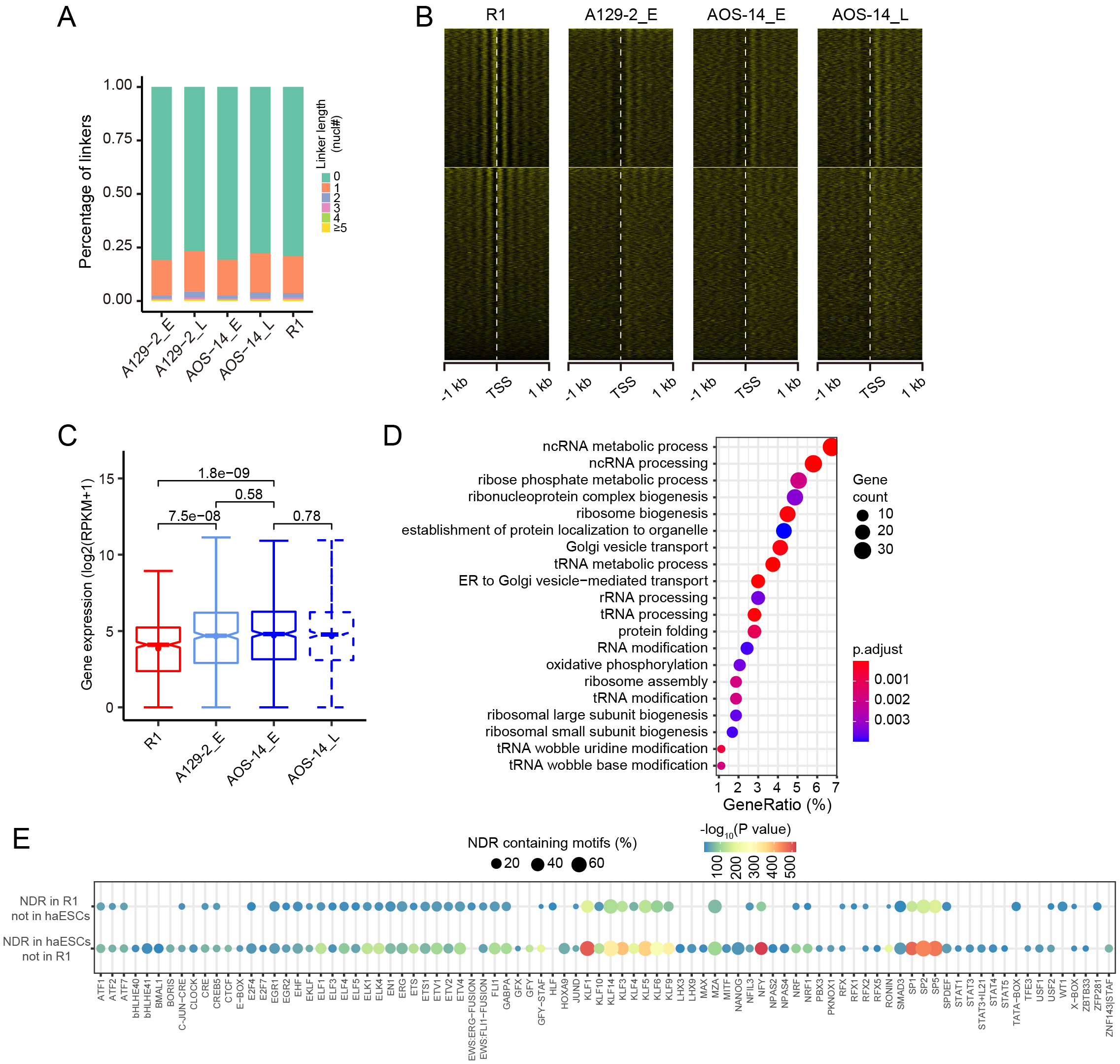


**Figure S2** Comparison of nucleosome organization between AG-haESCs and ESCs.

1. Percentage of linker DNA length categorized by the number of nucleosomes which a linker DNA can hold.
2. Heatmap showing the normalized nucleosome occupancy pattern around the transcription start sites (TSSs). The schematic diagram presenting the canonical arrangement of -1, NDR, +1, +2, +3 nucleosomes around the TSSs was summarized from all the transcripts. NDR: nucleosome depletion region.
3. Expression levels of the 1879 genes in Figure 2C (Wilcoxon test).
4. Functional annotation of the 2056 genes in Figure 2C.
5. Transcription factor motif enrichment in the promoter NDRs.


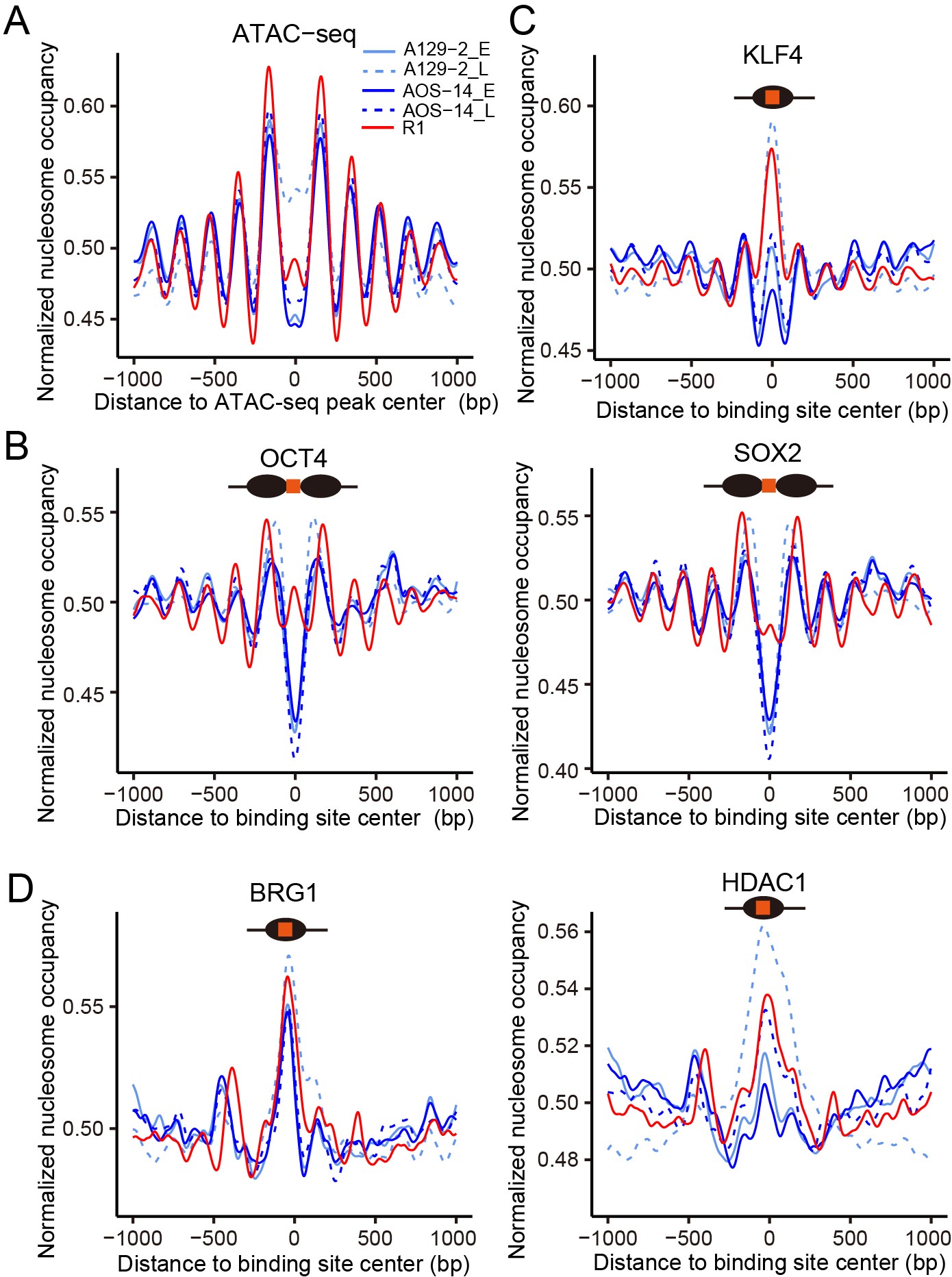


**Figure S3** Nucleosome distribution around the binding sites of representative factors.

1. The composite distribution of nucleosomes around ATAC-seq peaks.
2. The binding sites of the core pluripotency factors OCT4 and SOX2 preferentially reside in the linker region.
3. The binding sites of the core pluripotency factor KLF4 are predominantly present on nucleosomes.
4. The binding sites of the chromatin remodeling factor BRG1 and the histone deacetyltransferase HDAC1 are predominantly present on nucleosomes.


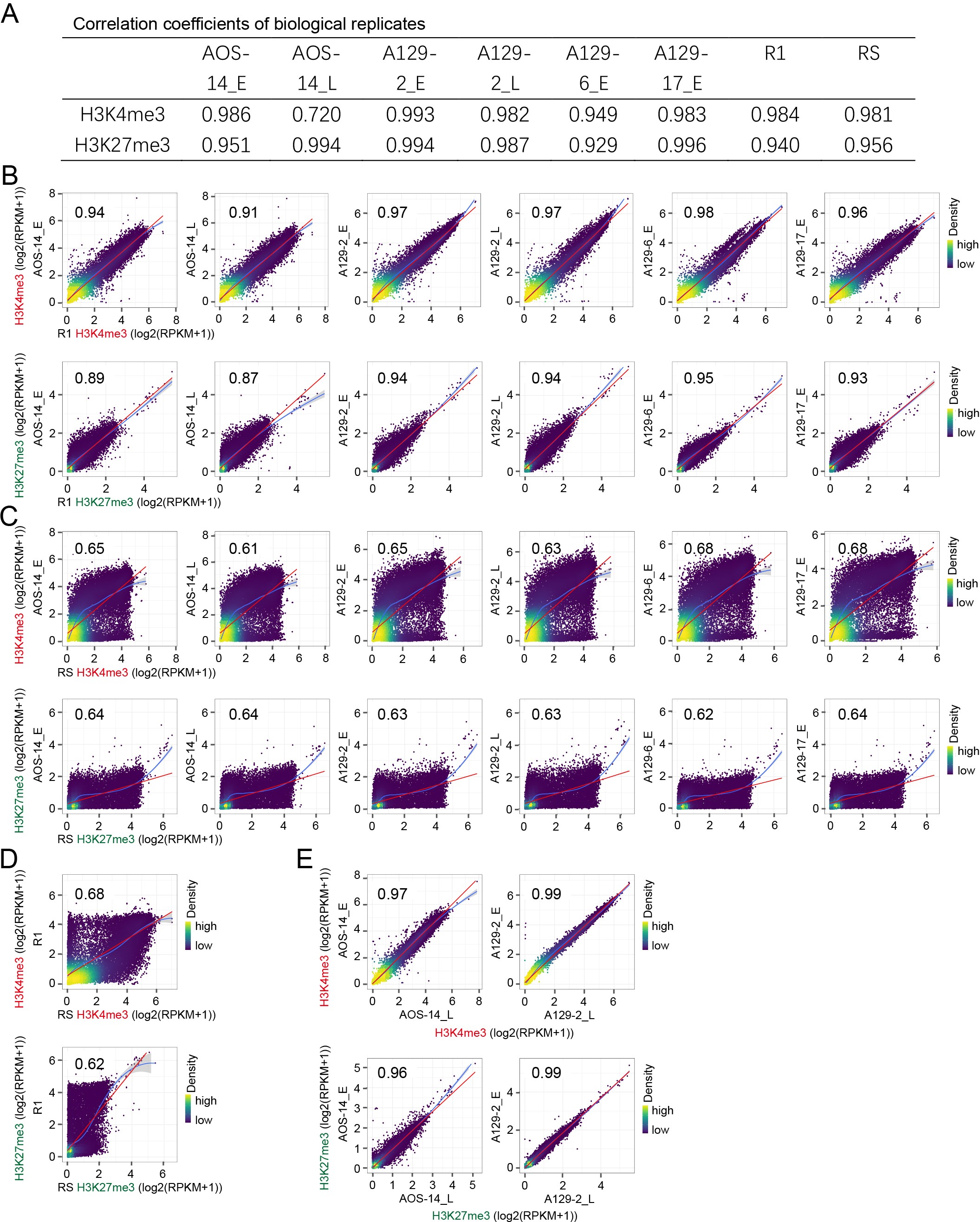


**Figure S4** Correlations of H3K4me3 and H3K27me3 signals in the promoters between AG-haESCs, ESC R1 and RS.

1. H3K4me3 and H3K27me3 ChIP-seq data are highly reproducible.
2. Histone modification signals in the promoters are highly similar between ESC R1 and AG-haESCs. Correlation coefficients are indicated.
3. Histone modification signals in the promoters are lowly correlated between round spermatids (RS) and AG-haESCs. Correlation coefficients are indicated.
4. Histone modification signals in the promoters are lowly correlated between round spermatids (RS) and ESC R1. Correlation coefficients are indicated.
5. Histone modification signals in the promoters are highly similar between

early- and late-passage AG-haESCs. Correlation coefficients are indicated. All correlations were calculated by Pearson test.


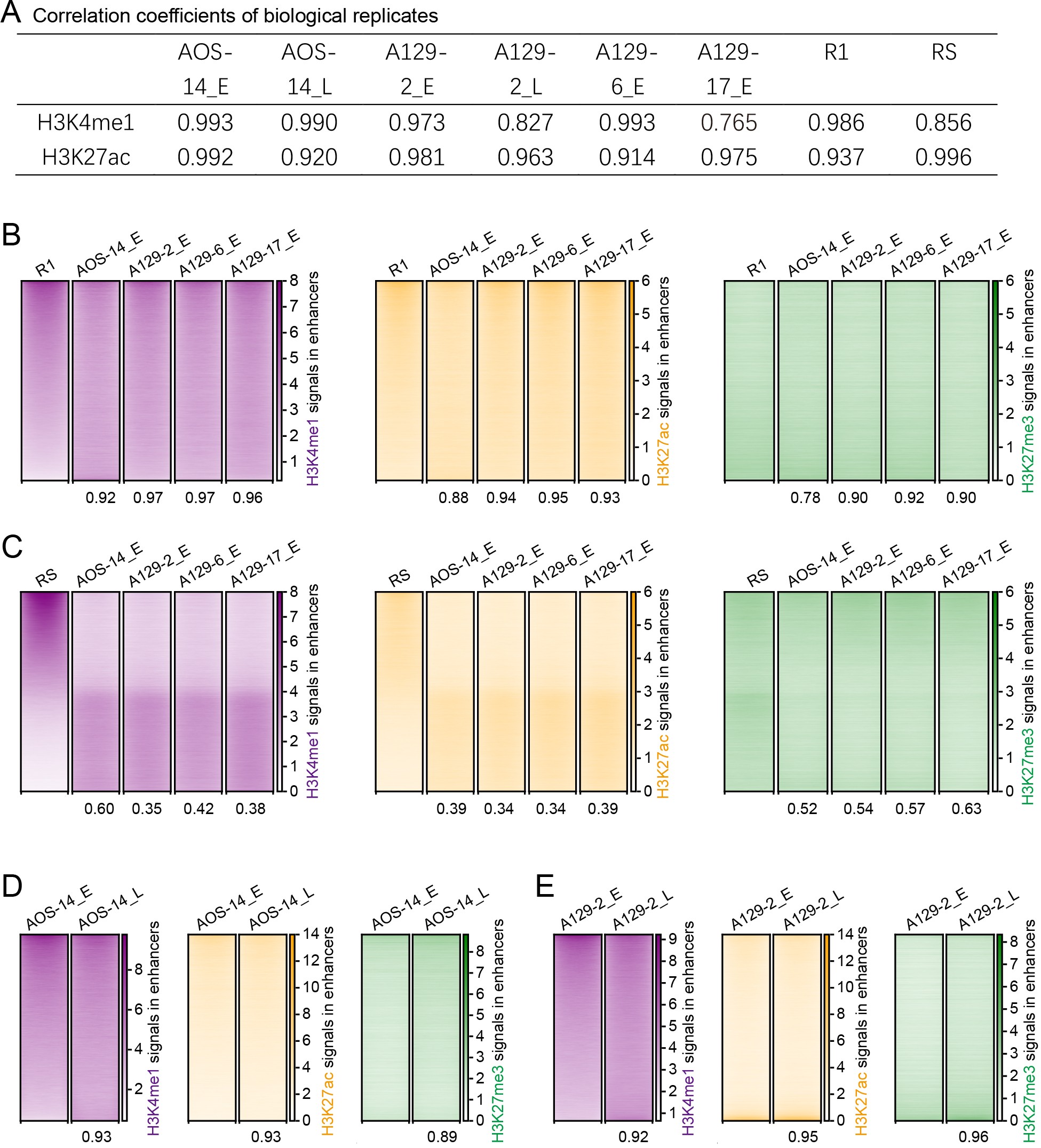


**Figure S5** Correlations of key histone modification signals in the enhancers between AG-haESCs, ESC R1 and round spermatids (RS).

1. H3K4me1 and H3K27ac ChIP-seq data are highly reproducible.
2. Histone modification signals in the enhancers are highly similar between ESC R1 and AG-haESCs. Correlation coefficients are indicated at bottom.
3. Histone modification signals in the enhancers are lowly correlated between round spermatids (RS) and AG-haESCs. Correlation coefficients are indicated at bottom.

D,E) Histone modification signals in the enhancers are highly similar between early- and late-passage AG-haESCs. Correlation coefficients are indicated at bottom.

All correlations were calculated by Pearson test.

References:

1. Zheng, P. and J. Dean, *Oocyte-specific genes affect folliculogenesis, fertilization, and early development.* Semin Reprod Med, 2007. **25**(4): p. 243-51.
